# Psychedelic assisted psychotherapy (2020–2025): a scoping review of clinical evidence, mechanisms, safety, and ethics

**DOI:** 10.3389/fpsyg.2026.1820860

**Published:** 2026-10-27

**Authors:** Nasiru Mohammed Abdullahi, Rashid Menhas, Patience Osose Nasir, Mohamed Goda Elbqry, Fatma Mohamed Elmansy, Saddam Ahmed Al-Ahdal, Ashwaq Hamad Alshmimry, Azza Elsayed Abdelfatah Arafat, Samia Eaid Elgazzar, Fatima S. O. Ashmieg

**Affiliations:** 1 Department of Community, Psychiatric and Mental Health Nursing, College of Nursing, Qassim University, Buraydah, Saudi Arabia; 2 Department of Nursing, Medical City, Qassim University, Buraydah, Saudi Arabia; 3 Department of Nursing Sciences, Faculty of Allied Health Sciences Ahmadu Bello University Zaria, Kaduna, Nigeria; 4 School of Nursing, Shandong Xiehe University, Jinan, China; 5 Department of Nursing, College of Applied Medical Sciences, Buraydah Private Colleges, Buraydah, Saudi Arabia; 6 Department of Nursing Science, Faculty of Applied Health Sciences, Edo State University, Iyamho, Nigeria; 7 Department of Medical-Surgical Nursing, College of Nursing, Qassim University, Buraydah, Saudi Arabia; 8 Department of Medical-Surgical Nursing, Faculty of Nursing, Suez Canal University, Ismailia, Egypt; 9 Department of Psychiatric and Mental Health Nursing, Zagazig University, Zagazig, Egypt; 10 Department of Medical-Surgical Nursing, Faculty of Nursing, Port-Said University, Port-Said, Egypt

**Keywords:** DMT, LSD, MDMA, psilocybin, psychedelic medicine, psychedelic-assisted therapy, psychotherapy

## Abstract

**Background:**

Clinical research on psychedelic-assisted therapy (PAT) has expanded substantially in recent years, particularly for post-traumatic stress disorder (PTSD) and depressive disorders. Contemporary studies have generally evaluated psychedelic administration within controlled therapeutic settings that include psychological support, preparation, and integration. However, variability in clinical response and methodological challenges have highlighted the importance of individual, relational, and contextual factors, often described as “set and setting,” in understanding therapeutic outcomes. The role of psychotherapy and the broader therapeutic context therefore remains an important area of investigation.

**Objective:**

This scoping review aimed to map the contemporary evidence on PAT, characterize the clinical and psychotherapeutic approaches used with psilocybin, MDMA, LSD, and DMT, summarize reported clinical and safety findings, and identify methodological, mechanistic, ethical, and implementation considerations across existing protocols.

**Methods:**

A structured search of PubMed, PsycINFO, Scopus, and ClinicalTrials.gov was conducted on 23 February 2026. Sources published or publicly available between January 2020 and November 2025 were eligible. The review included peer-reviewed and relevant non-peer-reviewed sources addressing psychedelic-assisted interventions, including primary clinical studies, reviews, clinical trial registrations, regulatory documents, and other relevant contextual sources. Records were managed using Zotero and screened against predefined Population, Concept, and Context criteria. Findings were synthesized descriptively according to source type and evidence domain.

**Results:**

The mapped evidence was most developed for MDMA-assisted therapy in PTSD and psilocybin-assisted interventions for depressive disorders. Randomized clinical trials reported reductions in PTSD and depressive symptoms following MDMA- and psilocybin-based interventions, respectively, while emerging clinical evidence also investigated LSD-based treatment for generalized anxiety disorder. Evidence for DMT-assisted therapy remained comparatively preliminary, with several available sources consisting of trial registrations or industry-reported information. Across the literature, preparation, supervised psychedelic administration, and integration were common therapeutic components, although the structure and intensity of psychotherapy varied substantially. Functional unblinding, expectancy effects, heterogeneity of psychotherapeutic approaches, and limitations in long-term safety data were recurring concerns. Ethical issues included informed consent during altered states of consciousness, therapist competence and boundaries, cultural considerations, and equitable access.

**Conclusion:**

PAT represents a rapidly developing area of clinical research, but the evidence base remains heterogeneous across compounds, indications, therapeutic models, and levels of evidential maturity. Future research should improve the standardization and reporting of psychotherapeutic interventions, strengthen methods for assessing blinding and expectancy, establish consistent long-term safety monitoring, and develop robust ethical and equity frameworks. Clear distinction between peer-reviewed clinical findings and preliminary or contextual evidence will also be essential as PAT research progresses toward broader clinical implementation.

## 1 Introduction

After decades of limited regulatory activity, interest in combining psychedelic compounds with structured psychotherapy has re-emerged as a major focus of psychiatric research. Recent phase 2 and 3 clinical trials have reported therapeutic benefits of psychedelic-assisted therapy (PAT) for several psychiatric conditions, although the evidence base remains heterogeneous and methodological limitations continue to complicate interpretation. Among the most advanced areas of research is MDMA-assisted therapy for post-traumatic stress disorder (PTSD). In a multisite phase 3 trial, participants receiving MDMA-assisted therapy showed significantly greater reductions in clinician-rated PTSD symptoms than those receiving therapy with placebo (Mitchell et al., 2023). Psilocybin has also demonstrated antidepressant effects in controlled clinical studies. In a randomized clinical trial, a single 25-mg dose administered with psychological support produced greater reductions in depressive symptoms than an active placebo, with benefits observed during follow-up (Raison et al., 2023).

Contemporary PAT protocols generally combine several stages of psychological support, including preparatory sessions, supervised drug administration, and subsequent integration sessions. These components are intended to establish therapeutic rapport, support participants during the acute psychedelic experience, and help them incorporate experiences or insights into everyday life. However, the specific nature, intensity, and theoretical orientation of psychotherapy vary considerably across studies. In MDMA-assisted therapy, for example, treatment protocols have incorporated a therapeutic approach emphasizing participant-led emotional processing and an “inner healing intelligence” framework. For classic psychedelics such as psilocybin and LSD, proposed therapeutic mechanisms include alterations in emotional processing, cognitive flexibility, self-related processing, and patterns of brain network activity. Associations between aspects of the acute subjective experience and subsequent clinical outcomes have also been reported, although these relationships do not by themselves establish causality.

Despite promising clinical findings, important methodological challenges remain. The pronounced subjective effects of many psychedelic compounds can compromise treatment blinding, allowing participants and therapists to infer treatment allocation. Functional unblinding may therefore contribute to expectancy effects and complicate the interpretation of both self-reported and clinician-rated outcomes. Public expectations surrounding psychedelic treatments may further influence participants’ experiences and treatment perceptions (Muthukumaraswamy et al., 2021; Aday et al., 2022). Regulatory guidance has consequently emphasized the importance of trial designs capable of more clearly characterizing the contributions of the investigational drug, psychotherapy, treatment context, and expectancy effects (US Food and Drug Administration, 2023).

Research on PAT has expanded beyond PTSD and depressive disorders to include anxiety disorders, substance use disorders, and psychological distress associated with serious or life-threatening illness. Emerging studies have also examined applications in other populations and therapeutic contexts, although the evidence remains preliminary in several of these areas (Back et al., 2024; Richard and Garcia-Romeu, 2025). Alongside the expanding clinical literature, ethical and practical questions have become increasingly important. These include the adequacy of informed consent in the context of altered states of consciousness, standards for therapist training and professional conduct, management of therapeutic boundaries, equitable access to treatment, and appropriate consideration of Indigenous knowledge and cultural contexts (Galderisi et al., 2024).

Amid this rapidly evolving clinical and regulatory landscape, this scoping review maps the evidence on psychedelic-assisted therapy involving psilocybin, MDMA, LSD, and DMT, with particular attention to clinical outcomes, proposed mechanisms, safety, therapeutic approaches, methodological challenges, and ethical considerations. It examines common principles and important differences across existing treatment protocols and identifies areas in which further research is needed to strengthen the evidence base and inform safe, equitable, and scientifically rigorous clinical development.

## 2 Methods

### 2.1 Review design and reporting

This study was conducted as a scoping review to map the available evidence on psychedelic-assisted therapy (PAT), characterize the types of evidence available, and identify major clinical, therapeutic, methodological, mechanistic, safety, and ethical considerations within the field. The review was informed by established scoping-review methodology and reported in accordance with the Preferred Reporting Items for Systematic Reviews and Meta-Analyses Extension for Scoping Reviews (PRISMA-ScR) guidelines (Tricco et al., 2018).

The review was structured using the Population, Concept, and Context (PCC) framework. This approach was selected because the objective was to map a broad and heterogeneous body of evidence rather than to estimate the comparative effectiveness of a specific intervention. Consistent with scoping-review methodology, the review considered multiple types of evidence sources, including primary research, secondary literature, clinical trial records, regulatory documents, and other relevant publicly available sources.

### 2.2 Eligibility criteria

Eligibility criteria were defined according to the PCC framework.

Population: The review considered evidence concerning human participants receiving, or being studied in relation to, psychedelic-assisted interventions. Relevant populations included individuals with psychiatric or psychological conditions such as major depressive disorder, treatment-resistant depression, post-traumatic stress disorder (PTSD), anxiety disorders, and substance use disorders. Evidence involving healthy participants was also considered where it provided relevant information on therapeutic mechanisms, safety, expectancy, psychotherapy, or methodological issues related to PAT.

Concept: The concept of interest was psychedelic-assisted therapy involving psilocybin, MDMA, LSD, or DMT in conjunction with psychotherapy, psychological support, preparation, integration, or related therapeutic procedures. Evidence addressing clinical outcomes, therapeutic models, safety and adverse events, mechanisms of action, methodological challenges, expectancy and blinding, and ethical considerations was also considered.

Context: The review encompassed clinical and research settings in which psychedelic-assisted interventions were investigated or delivered, as well as relevant regulatory and therapeutic-development contexts. This included structured psychotherapy settings, clinical trials, ongoing registered studies, regulatory frameworks, and other sources documenting the development or implementation of PAT.

Sources published or publicly available between January 2020 and November 2025 were eligible. The review included both peer-reviewed and relevant non-peer-reviewed sources to capture the breadth of evidence in this rapidly developing field. Peer-reviewed sources included primary clinical studies, systematic reviews, meta-analyses, narrative and mechanistic reviews, and other substantive scholarly publications. Non-peer-reviewed sources included clinical trial registry records, regulatory documents, organizational reports, and company communications when they provided relevant information concerning ongoing research, trial design, regulatory developments, treatment protocols, or preliminary findings not yet available in peer-reviewed publications.

Sources were excluded when they focused exclusively on animal or preclinical research; examined pharmacological, molecular, or neurobiological effects without relevance to therapeutic psychedelic use; addressed recreational or non-clinical psychedelic use; were editorials, opinion pieces, news items, or commentaries without substantive relevance to the review objectives; were published in languages other than English; or fell outside the specified publication period.

Importantly, source inclusion did not imply equivalent evidential weight. During data charting and synthesis, sources were classified according to their type and evidential role. Peer-reviewed primary clinical studies were distinguished from secondary literature, registered clinical trials, regulatory documents, and industry-reported or other preliminary information. Findings from non-peer-reviewed sources were therefore presented as contextual, regulatory, ongoing, or preliminary evidence, as appropriate, rather than as established clinical findings.

### 2.3 Information sources

The literature search was conducted using PubMed, PsycINFO, and Scopus to identify scholarly publications relevant to PAT. ClinicalTrials.gov was searched separately to identify registered and ongoing clinical studies and to capture information on the developing clinical research landscape.

Additional sources were identified through manual screening of the reference lists of included publications and relevant reviews. Regulatory and other publicly available sources identified during the search and reference-screening processes were also considered where they met the predefined eligibility criteria.

The selection of multiple source types was intentional because scoping reviews are suited to mapping heterogeneous evidence, including published research and relevant non-research sources, particularly in emerging fields where important developments may precede peer-reviewed publication.

### 2.4 Search strategy

Search strategies were developed using combinations of controlled vocabulary and free-text terms relating to psychedelic-assisted therapy, the compounds of interest (psilocybin, MDMA, LSD, and DMT), psychotherapy and psychological support, psychiatric and psychological conditions, clinical research, safety, mechanisms, and ethical considerations.

The searches were conducted on 23 February 2026. The date of the database search was distinct from the eligibility period, which was limited to sources published or publicly available from January 2020 through November 2025.

Database-specific search strings, search fields, Boolean operators, filters, and other search limits are provided in Supplementary Table S1. The search strategies were adapted to the indexing system and syntax of each database. Reference lists of included studies and relevant reviews were subsequently screened to identify additional eligible sources. The detailed reporting of the search strategy was intended to facilitate reproducibility, consistent with recommendations for scoping-review searches.

### 2.5 Study selection

All records retrieved from the electronic searches were imported into Zotero reference manager, where duplicate records were identified and removed. The remaining records were screened by title and abstract against the predefined eligibility criteria. Potentially relevant records were subsequently assessed at the full-text or complete-record level, depending on the source type.

Two reviewers independently screened records during the title/abstract and full-text stages. Disagreements were resolved through discussion and consensus. Reasons for exclusion at the full-text stage were documented and summarized in the PRISMA-ScR flow diagram (Figure 1).

**Figure 1:**
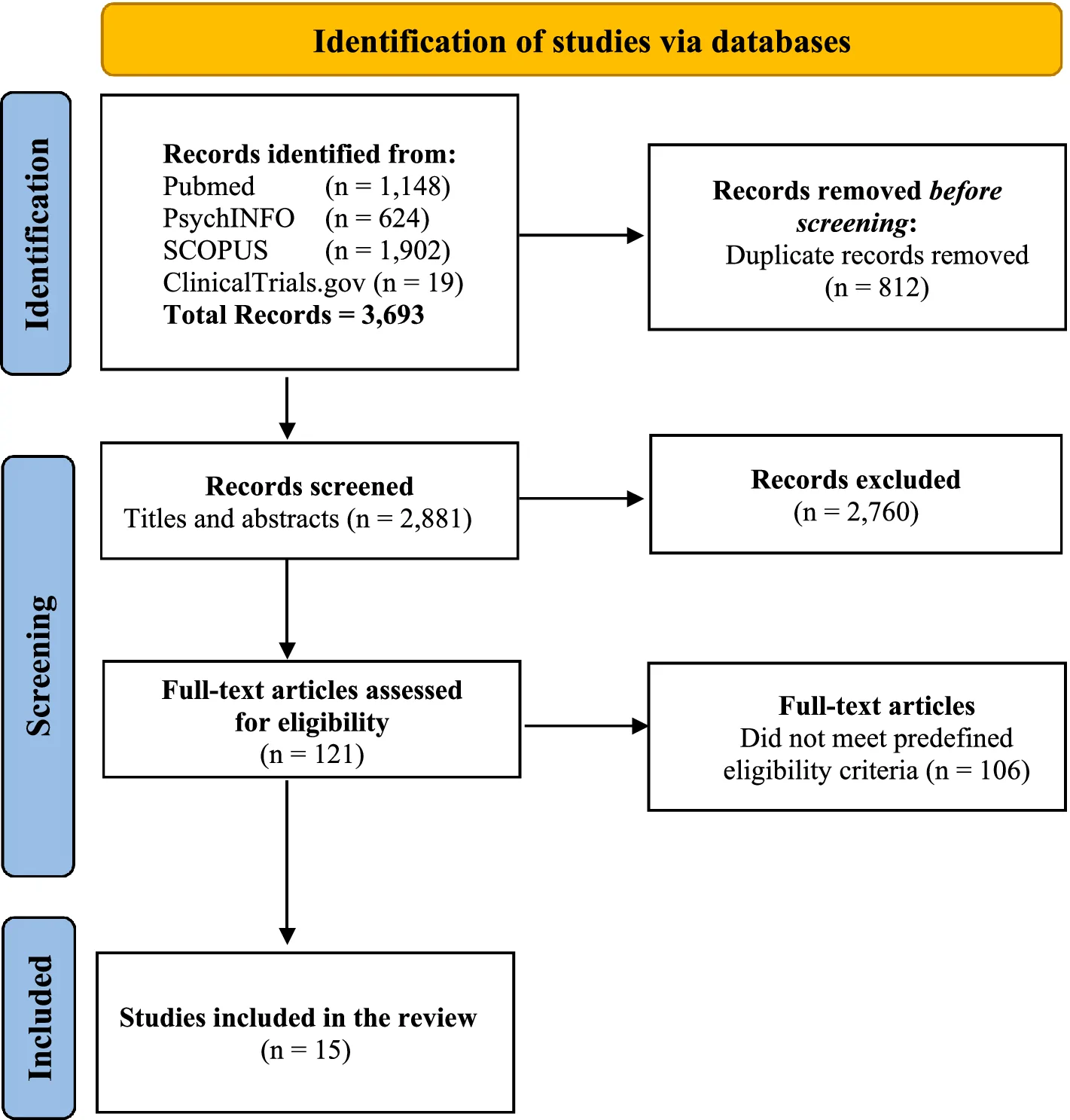
PRISMA flowchart of the scoping review process on Psychedelic Assisted Psychotherapy (2020–2025).

Because the review included different types of evidence sources, the selection process also recorded the source category of each included record. These categories included primary clinical studies, secondary scholarly literature, clinical trial registrations, regulatory documents, and industry or organizational sources.

### 2.6 Data charting

Data were charted using a standardized approach. Information extracted from primary clinical studies included:

- author and year;
- study design and phase, where applicable;
- sample size and participant characteristics;
- psychedelic compound and dose;
- psychotherapy or psychological-support protocol;
- clinical indication;
- comparator or control condition;
- treatment setting;
- duration and follow-up;
- primary and relevant secondary outcomes;
- adverse events and safety findings; and.
- major methodological limitations.

For reviews and other secondary scholarly publications, data included the review type, scope, principal findings, and relevance to clinical, mechanistic, methodological, therapeutic, safety, or ethical questions.

For clinical trial registrations, regulatory documents, and industry or organizational sources, information was charted according to the purpose of the source, including intervention characteristics, trial design, regulatory considerations, study status, preliminary findings, or other relevant contextual information.

The source type and publication status were recorded for each included source to ensure that peer-reviewed clinical evidence could be distinguished from preliminary or contextual information during synthesis.

### 2.7 Evidence synthesis

Given the heterogeneity of the included sources, interventions, therapeutic approaches, clinical populations, outcomes, and evidence types, findings were synthesized descriptively rather than through quantitative meta-analysis.

The evidence was organized into six broad domains:

1. clinical outcomes and therapeutic effects;
2. psychotherapy and therapeutic models;
3. methodological considerations, including blinding and expectancy;
4. mechanisms of action;
5. safety and adverse events; and.
6. ethical, regulatory, and implementation considerations.

Primary clinical studies were presented separately from secondary literature and contextual sources. Clinical trial registrations, regulatory documents, and industry-reported findings were explicitly identified as such and were not treated as equivalent to peer-reviewed clinical results.

Where a secondary publication discussed primary studies that were also included in the review, the secondary source was used to provide context or interpretation rather than being counted as an additional independent clinical observation. This approach was used to minimize duplication and avoid overstating the amount of available clinical evidence.

No quantitative meta-analysis, formal risk-of-bias assessment, or GRADE assessment was undertaken. The purpose of the synthesis was to map the breadth and characteristics of the available evidence, identify areas of convergence and uncertainty, and highlight methodological, clinical, safety, ethical, and regulatory issues requiring further investigation. Key methodological, ethical, safety, and implementation findings identified across the evidence are summarized in Supplementary Table S2.

### 2.8 Ethical considerations

As this study involved the synthesis of previously published or publicly available information and did not involve direct recruitment or interaction with human participants, institutional ethical approval was not required.

## 3 Results

### 3.1 Overview of the evidence base

The evidence identified for the period January 2020 to November 2025 encompassed psychedelic-assisted interventions involving MDMA, psilocybin, LSD, and DMT across several psychiatric indications, particularly post-traumatic stress disorder (PTSD), major depressive disorder (MDD), treatment-resistant depression (TRD), and anxiety disorders. The evidence base comprised randomized clinical trials, open-label studies, follow-up analyses, reviews, methodological publications, clinical trial registrations, regulatory documents, and other contextual sources. These sources were considered separately according to their evidence type during data charting and synthesis.

The most developed clinical evidence concerned MDMA-assisted therapy for PTSD and psilocybin-assisted therapy for depressive disorders. Evidence concerning LSD- and DMT-assisted interventions was comparatively limited and included both peer-reviewed clinical studies and preliminary or developmental sources. Across the clinical studies, psychedelic administration was generally accompanied by psychological support or psychotherapy, although the structure, intensity, duration, and theoretical orientation of the therapeutic component varied between studies. The characteristics and evidence types of the principal sources are summarized in Table 1.

**Table 1:** Summary of clinical trials and reviews on psychedelic-assisted therapy (2020–2025).

Authors/Year	Study type	Compound/focus	Indication/topic	Sample size	Dose	Therapy model	Primary outcome	Effect size	Adverse events	Follow-up
Mitchell et al. (2021)	Clinical Trial	MDMA	PTSD	90	75–125 mg (2–3 sessions)	Manualized trauma-focused	CAPS-5 reduction	d ≈ 0.91	Mild, transient	12 months
Mitchell et al. (2023)	Clinical Trial	MDMA	PTSD	104	75–125 mg (2–3 sessions)	Manualized trauma-focused	CAPS-5 LS mean change −23.7 vs. − 14.8	Large	Mild, transient	12 months
Raison et al. (2023)	Clinical Trial	Psilocybin	MDD	104	25 mg (single dose)	Structured psychological support	MADRS reduction	Rapid, sustained	Mild, transient	6 weeks
Goodwin et al. (2022)	Clinical Trial	Psilocybin	TRD	233	1 mg, 10 mg, or 25 mg	Structured psychological support	MADRS change	Dose–response	Mild, transient	12 weeks
COMPASS Pathways (2022)	Clinical Trial	Psilocybin	TRD follow-up	Subset	25 mg	Structured psychological support	Maintenance of benefit	N/A	Mild, transient	52 weeks
Robison et al. (2025)	Clinical Trial	LSD (MM120)	GAD	198	100 μg (single dose)	Supportive therapy	HAM-A reduction	Clinically meaningful	Mild, transient	4 weeks
Small Pharma Inc. and Cybin Inc. (2023)	Clinical Trial	DMT (SPL026)	MDD	34	Single IV dose	Supportive therapy	MADRS reduction	Rapid onset	Mild, transient	12 weeks
Aday et al. (2025)	Open-label pilot trial	Psilocybin	Fibromyalgia	5	15 mg & 25 mg (2 doses)	Psilocybin-assisted therapy	Exploratory clinical outcomes	N/A	Transient cardiovascular changes/ headache	4 weeks
Rosenbaum et al. (2024)	Riak-benefit analysis	PAT	Therapist training	N/A	N/A	Experimental training	Training-related risks and benefits	N/A	N/A	N/A
Carhart-Harris et al. (2021)	Primary randomized trial	Psilocybin	MDD	59	Two 25-mg doses vs. 1-mg control	Psilocybin+ psychological support	QIDS-SR-16 and secondary outcomes	Primary outcome	Not statistically significant	6 weeks
Vollenweider and Smallridge (2022)	Mechanistic Review	Classic psychedelics	Therapeutic mechanisms	N/A	N/A	N/A	Scope: cognitive flexibility	N/A	N/A	N/A
James et al. (2024a)	Mechanistic Study	DMT	Pharmacodynamics	N/A	N/A	N/A	Scope: tolerability	N/A	N/A	N/A
James et al. (2024b)	Open-Label Study	Drug interactions	SPL026 + SSRIs	13	N/A	N/A	Scope: safety and efficacy	N/A	N/A	N/A
US Food and Drug Administration (2023)	Regulatory Guidance	Psychedelics	Trial design considerations	N/A	N/A	N/A	Scope: safety monitoring	N/A	N/A	N/A
Contreras (2023)	Regulatory Guidance	Psychedelics	Data standards	N/A	N/A	N/A	Scope: conduct standards	N/A	N/A	N/A

### 3.2 PTSD: MDMA-assisted therapy

Two multicenter phase 3 randomized trials evaluated MDMA-assisted therapy for PTSD. The first trial included 90 participants with severe PTSD who were randomized to MDMA-assisted therapy or placebo with manualized psychotherapy. MDMA-assisted therapy produced a significantly greater reduction in clinician-administered PTSD symptoms than placebo-assisted therapy, with a between-group effect size of 0.91 for the primary CAPS-5 outcome. Functional impairment, assessed using the Sheehan Disability Scale, also improved significantly in the MDMA group (Mitchell et al., 2021).

A subsequent phase 3 trial included 104 participants with moderate to severe PTSD and replicated the primary finding. The least-squares mean change in CAPS-5 score was −23.7 with MDMA-assisted therapy compared with −14.8 with placebo plus therapy (*p* < 0.001; Cohen’s d = 0.7). Improvement in functional impairment was also greater in the MDMA group, although the effect was smaller than that observed for the primary PTSD outcome (Mitchell et al., 2023). Seven severe treatment-emergent adverse events were reported across the two treatment groups, with no serious treatment-emergent adverse events or deaths during the trial.

### 3.3 MDD and TRD: psilocybin-assisted therapy

Clinical studies of psilocybin-assisted therapy included both MDD and TRD populations. In a phase 2 randomized clinical trial involving 104 adults with MDD, a single 25-mg dose of psilocybin administered with psychological support was associated with rapid reductions in depressive symptoms, with treatment effects observed during follow-up through 6 weeks (Raison et al., 2023).

In a larger randomized trial involving 233 participants with TRD, participants received a single 1-, 10-, or 25-mg dose of psilocybin with psychological support. The 25-mg group demonstrated greater improvement in depressive symptoms at 3 weeks than the 1-mg group, providing evidence of a dose-related difference in the primary efficacy outcome (Goodwin et al., 2022).

The evidence also included the phase 2 randomized trial comparing psilocybin with escitalopram in 59 participants with MDD. Participants received two doses of psilocybin or a low-dose psilocybin control together with psychological support and 6 weeks of escitalopram or placebo. The primary between-group comparison on the QIDS-SR-16 at 6 weeks did not reach statistical significance, although several secondary outcomes favored psilocybin (Carhart-Harris et al., 2021). This study was therefore classified as a primary clinical trial rather than a mechanistic review.

Longer-term follow-up data were also identified from psilocybin development programs. These analyses provided information on persistence of treatment response beyond the primary trial endpoints but were based on subsets or follow-up cohorts and were therefore interpreted separately from the randomized primary analyses.

### 3.4 Anxiety disorders: LSD-based therapy

Evidence for LSD-based treatment was less extensive than that for MDMA and psilocybin. A phase 2b multicenter randomized, double-blind, placebo-controlled trial evaluated a single dose of MM120 (lysergide) in 198 adults with moderate to severe generalized anxiety disorder. Participants were randomized to placebo or 25-, 50-, 100-, or 200-μg doses. The study identified a significant dose-related reduction in anxiety symptoms, with the 100-μg dose producing a substantial improvement at the primary four-week endpoint (Robison et al., 2025).

The trial therefore provided peer-reviewed clinical evidence supporting further investigation of single-dose lysergide for generalized anxiety disorder. However, interpretation remains subject to the characteristics of the selected study population, the single-dose design, and the methodological challenges associated with maintaining blinding in psychedelic trials.

### 3.5 DMT-assisted therapy

The evidence concerning DMT-assisted therapy was comparatively preliminary. The sources identified during the review included early-phase clinical research, trial-registration records, and industry-reported information concerning investigational formulations such as SPL026. These sources described the development of DMT-based interventions for depressive disorders, including studies examining single-dose administration with psychological support.

Because a substantial proportion of the available DMT evidence consisted of trial registrations or company-reported information rather than completed peer-reviewed clinical publications, these findings were classified as preliminary or contextual evidence. Consequently, claims concerning efficacy, durability, safety, or drug interactions were not treated as equivalent to findings from completed peer-reviewed randomized clinical trials.

Source verification also identified citation discrepancies in the previously assembled evidence base. In particular, Luca et al. (2024) did not provide evidence concerning DMT or SPL026 and were therefore excluded from the DMT synthesis. The publication concerns a phase 1 investigation of PNT001, an anti-tau antibody, rather than a psychedelic intervention.

### 3.6 Methodological and regulatory findings

Difficulty maintaining treatment blinding emerged as a recurring methodological issue across the psychedelic literature. The pronounced subjective effects of psychedelic compounds may enable participants and therapists to infer treatment allocation, creating the potential for expectancy and performance effects. Methodological literature identified several approaches to addressing this problem, including more credible active comparators, prospective assessment of treatment-guessing and expectancy, and improved reporting of blinding integrity.

The review also identified literature examining the role of psychotherapy in PAT. The available publications described considerable variation in the amount, structure, and theoretical orientation of psychological support provided before, during, and after psychedelic administration. Aday et al. (2024), for example, highlighted the relatively limited empirical literature examining psychotherapy as a distinct component of psychedelic-assisted treatment and emphasized the need for greater attention to psychotherapy processes and therapeutic models.

Regulatory sources identified during the review similarly emphasized methodological and safety considerations specific to psychedelic clinical trials. These included the effects of psychoactive treatment on blinding, appropriate safety monitoring, characterization of the intervention and therapeutic context, and the need for rigorous trial conduct and outcome assessment. Regulatory documents were treated as contextual evidence and were not used as evidence of clinical efficacy.

### 3.7 Mechanistic evidence and psychotherapy components

The mechanistic literature identified several proposed pathways through which classic psychedelics may contribute to therapeutic change. These included serotonin 5-HT2A receptor signaling, alterations in large-scale brain-network activity, changes in cognitive and emotional processing, and increased psychological flexibility. Reviews of classic psychedelics have described these mechanisms as interacting processes rather than as a single established pathway.

The therapeutic interventions accompanying psychedelic administration also varied considerably. Common elements included preparatory sessions before dosing, monitored support during the acute psychedelic experience, and post-session integration. However, the specific content, duration, therapist orientation, and degree of structure differed between studies.

For MDMA-assisted therapy, the therapeutic model was more explicitly standardized across the phase 3 program, whereas classic psychedelic studies showed greater variation in the nature of psychological support. The literature also identified therapist-related factors, therapeutic alliance, participant expectations, and the management of suggestibility as areas requiring further empirical investigation. At present, however, the available evidence does not establish that any single psychotherapeutic model or therapist-related factor is necessary or sufficient for clinical improvement.

### 3.8 Safety and adverse events

Across the contemporary clinical studies identified in the review, commonly reported acute adverse events included headache, nausea, dizziness, transient anxiety, and temporary physiological changes such as elevations in blood pressure. The frequency and severity of adverse events varied according to compound, dose, clinical population, and study design.

The safety findings were obtained predominantly from controlled clinical settings involving participant screening, preparation, supervised administration, and post-session monitoring. Consequently, the observed safety profiles reflect the characteristics of these selected research populations and treatment conditions. The absence of a particular adverse event in individual studies was not interpreted as evidence that the event could not occur in other populations or under less controlled conditions.

Similarly, the available evidence was insufficient to establish the absence of longer-term psychiatric or cardiovascular risks. Many clinical trials excluded participants with conditions considered to increase the risk of adverse outcomes, and follow-up periods varied substantially between studies. Safety findings were therefore interpreted in relation to sample size, participant selection, duration of follow-up, and study design rather than as definitive estimates of population-level risk.

Challenging psychological experiences, including transient anxiety, fear, perceptual disturbances, or emotional distress, were also reported across the literature. Some publications proposed that appropriately supported challenging experiences may contribute to therapeutic processes. However, this interpretation remains provisional, and challenging experiences were not considered inherently therapeutic in the present synthesis.

### 3.9 Ethical findings

Ethical considerations emerged as a recurring theme across the literature on psychedelic-assisted therapy. The reviewed sources identified several issues associated with the vulnerability of participants during altered states of consciousness and the intensive interpersonal nature of psychedelic-assisted treatment. Informed consent was therefore identified as an important consideration, particularly because participants may experience substantial changes in perception, cognition, emotional processing, and suggestibility during psychedelic administration.

The literature also emphasized therapist competence, professional conduct, and the maintenance of appropriate therapeutic boundaries. Particular attention was given to the potential vulnerability of participants to interpersonal influence during psychedelic experiences, including the ethical management of touch and other forms of physical or relational contact (McGuire et al., 2024; Aicher et al., 2025; McHerron et al., 2025). These findings highlighted the importance of appropriate therapist training, clearly defined professional boundaries, and procedures for responding to psychological distress (McHerron et al., 2025).

Cultural and equity considerations were also identified. The reviewed literature raised questions concerning the representation of diverse populations in psychedelic research, equitable access to emerging treatments, and the appropriate consideration of Indigenous knowledge and cultural contexts. Barriers related to treatment cost, availability of trained therapists, geographic access, and reimbursement were identified as potential factors affecting equitable implementation. Overall, the ethical findings indicated that safe translation of psychedelic-assisted therapy into broader clinical practice requires attention not only to pharmacological safety but also to informed consent, therapist competence, interpersonal boundaries, cultural context, and equitable access.

## 4 Discussion

This scoping review mapped the rapidly expanding literature on psychedelic-assisted therapy (PAT) published or publicly available between 2020 and 2025. The evidence base varied substantially across compounds, clinical indications, study designs, and levels of evidential maturity. Peer-reviewed clinical evidence was most developed for MDMA-assisted therapy in PTSD and psilocybin-assisted interventions for depressive disorders, whereas the evidence for LSD- and DMT-assisted interventions was comparatively less extensive. The review also identified recurring methodological, psychotherapeutic, safety, ethical, and regulatory issues that influence interpretation of the emerging clinical literature.

### 4.1 Clinical evidence across psychedelic-assisted interventions

The phase 3 MDMA trials provided relatively substantial controlled evidence concerning MDMA-assisted therapy for PTSD. Both trials reported greater reductions in clinician-rated PTSD symptoms with MDMA-assisted therapy than with placebo-assisted therapy, accompanied by improvements in functional outcomes (Mitchell et al., 2021, 2023). These findings are important because the trials used structured psychotherapy in both treatment conditions, allowing the clinical comparison to reflect the addition of MDMA within a defined therapeutic framework rather than a comparison between drug treatment and psychotherapy alone.

At the same time, the findings should not be interpreted independently of the methodological characteristics of psychedelic trials. The marked subjective effects of MDMA can compromise treatment masking and increase the likelihood that participants and therapists infer allocation. Consequently, expectancy and other nonspecific effects may contribute to observed between-group differences. Recent evidence syntheses have similarly identified concerns relating to blinding, heterogeneity of psychotherapy, and study quality within the MDMA literature.

The psilocybin literature showed a similarly expanding but heterogeneous evidence base. Randomized trials in MDD and TRD reported relatively rapid reductions in depressive symptoms following single-dose administration with psychological support (Goodwin et al., 2022; Raison et al., 2023). However, results differed according to comparator, outcome measure, follow-up period, and study population. The trial comparing psilocybin with escitalopram, for example, did not demonstrate a statistically significant between-group difference on its prespecified primary outcome at 6 weeks, despite favorable findings on several secondary outcomes (Carhart-Harris et al., 2021). These differences highlight the importance of considering the complete outcome structure of individual trials rather than describing the psilocybin evidence as uniformly positive.

The evidence for LSD-assisted treatment was more limited but included a randomized phase 2b trial of MM120 in generalized anxiety disorder. The reported reduction in anxiety symptoms following a single administration provides a basis for continued clinical investigation, although the evidence remains constrained by the characteristics of the trial population, single-dose design, follow-up duration, and difficulties with maintaining treatment masking (Robison et al., 2025).

DMT-assisted therapy remained at an earlier stage of development during the review period. Much of the available information concerning SPL026 originated from trial registrations and industry communications rather than completed peer-reviewed clinical publications. These sources are useful for mapping the development of the field, but do not provide the same evidential basis as independently published clinical results. Accordingly, claims regarding the efficacy, durability, safety, or interaction profile of DMT-based interventions should remain provisional until supported by adequately reported peer-reviewed clinical studies.

### 4.2 The psychotherapeutic component of PAT

A central finding across the mapped literature was that PAT is not simply a pharmacological intervention. Most contemporary clinical protocols incorporate some form of preparation, support during psychedelic administration, and post-session integration. However, the nature and intensity of these components vary considerably between studies.

This variability creates an important challenge for interpreting clinical outcomes. When psychedelic administration and psychotherapy are delivered together, the observed treatment effect may reflect pharmacological effects, therapeutic processes, expectancy, the treatment environment, or interactions among these components. The available literature does not yet establish the relative contribution of each factor.

The evidence concerning psychotherapy itself also remains comparatively underdeveloped. Therapeutic approaches range from supportive and relatively nondirective models to more structured or integrative approaches. Differences have been reported in therapist training, session structure, preparation, integration, management of acute experiences, and the use of therapeutic techniques (Aday et al., 2024) similarly highlighted the limited empirical investigation of psychotherapy as a distinct component of psychedelic-assisted treatment. Further research should therefore evaluate psychotherapy processes explicitly rather than treating psychological support as a uniform intervention across trials.

Claims concerning particular therapeutic models should also be interpreted cautiously. The present evidence does not establish that directive approaches are superior to nondirective approaches, nor that a particular therapist stance or integration model is necessary for therapeutic benefit. Comparative studies that manipulate or systematically characterize these components are needed.

### 4.3 Mechanisms of therapeutic action

The mechanistic literature provides several plausible explanations for how psychedelic compounds may contribute to therapeutic change. Classic psychedelics such as psilocybin and LSD primarily exert their acute effects through serotonergic mechanisms involving the 5-HT2A receptor, with downstream changes in brain-network activity, perception, cognition, and emotional processing (Vollenweider and Smallridge, 2022). Proposed psychological mechanisms include increased cognitive flexibility, modification of maladaptive beliefs or priors, enhanced emotional processing, and changes in self-related processing.

These mechanisms should nevertheless be distinguished from demonstrated mediators of clinical outcome. Associations between the intensity or characteristics of the acute psychedelic experience and subsequent improvement do not establish that the experience itself causes therapeutic change. Expectancy, therapeutic alliance, contextual factors, and other components of the treatment environment may contribute simultaneously.

MDMA differs pharmacologically from classic serotonergic psychedelics and has been investigated in relation to emotional processing, social connection, fear responses, and trauma-related psychotherapy. Its effects may therefore interact with the therapeutic processes used in PTSD treatment. However, the relative contributions of pharmacological and psychotherapeutic mechanisms remain incompletely characterized.

### 4.4 Methodological challenges: blinding and expectancy

Functional unblinding emerged as one of the most important methodological issues across the evidence base. The recognizable subjective effects of psychedelic compounds make conventional placebo-controlled designs difficult to maintain. Participants who correctly infer their treatment allocation may subsequently report outcomes differently, while therapists who know or infer allocation may alter their interactions with participants.

These concerns do not invalidate randomized trials, but they complicate interpretation of their effect estimates. Future studies should therefore incorporate prospective assessment of treatment-guessing, expectancy, and masking integrity. The development of more credible active comparators may also improve the interpretability of controlled trials, although no comparator completely reproduces the complex subjective and physiological effects of psychedelic drugs.

The regulatory literature has similarly recognized these challenges. The FDA’s 2023 draft guidance on psychedelic drug development specifically highlighted the distinctive effects of psychedelics on consciousness and the need to account for these characteristics in clinical trial design, including the role of psychotherapy, safety monitoring, dose–response assessment, and durability of treatment effects.

### 4.5 Safety and long-term monitoring

The contemporary clinical trials reviewed generally reported acute adverse events such as headache, nausea, dizziness, transient anxiety, and temporary cardiovascular changes. However, these findings should be interpreted in the context of intensive participant screening, controlled administration, trained supervision, and exclusion of individuals considered to be at elevated risk for certain psychiatric or medical complications.

Accordingly, the absence of particular serious outcomes in individual trials should not be interpreted as evidence that those risks are absent. Sample sizes remain relatively small for several compounds, follow-up durations vary, and trial populations may not represent the full range of patients who could eventually receive these interventions. A precise estimate of the frequency of rare serious adverse events also cannot be derived reliably by simply pooling heterogeneous studies without prespecified definitions and denominators.

Longitudinal pharmacovigilance will therefore be important as psychedelic interventions progress into larger and more diverse populations. Future research should use standardized adverse-event definitions, consistent reporting procedures, systematic assessment of psychiatric and cardiovascular outcomes, and sufficiently long follow-up to identify delayed or uncommon events.

Challenging psychological experiences require similar caution. Anxiety, fear, perceptual disturbances, or emotional distress during the acute psychedelic state may occur even under controlled conditions. Some authors have proposed that such experiences can form part of a therapeutic process when appropriately supported, but this remains an interpretive hypothesis rather than evidence that adverse experiences are intrinsically beneficial.

### 4.6 Ethical and clinical implementation considerations

The transition from controlled research environments to broader clinical implementation raises ethical issues that extend beyond pharmacological safety. Participants undergoing altered states of consciousness may experience increased psychological vulnerability and heightened sensitivity to interpersonal and environmental cues. This places particular importance on informed consent, therapist competence, professional boundaries, appropriate management of touch and interpersonal contact, and clear procedures for responding to distress.

Therapist training is therefore likely to be an important component of safe implementation. However, the optimal content and duration of training remain incompletely defined. Training frameworks should be informed by empirical evidence concerning therapist competence, therapeutic alliance, management of acute psychological experiences, and integration rather than relying solely on consensus or professional tradition.

Equity is another important consideration. Existing clinical trials may not adequately represent the demographic and socioeconomic diversity of the populations who could ultimately seek PAT. Barriers related to cost, treatment availability, geographic access, therapist training, and reimbursement could affect who is able to benefit from emerging interventions. Future research and implementation frameworks should therefore consider representativeness during recruitment and accessibility during clinical translation.

Ethical implementation also requires attention to cultural context and Indigenous knowledge where relevant to the development and use of psychedelic practices. Such considerations should avoid treating Indigenous traditions simply as sources of therapeutic techniques while overlooking questions of cultural ownership, representation, benefit sharing, and historical context.

### 4.7 Regulatory, research-integrity, and phase 3 developments

Developments occurring toward the end of the review period further illustrate the importance of interpreting psychedelic-assisted therapy within its regulatory and research-integrity context. In 2024, the U.S. Food and Drug Administration issued a Complete Response Letter concerning the MDMA-assisted therapy application for PTSD. The regulatory response required deficiencies in the application to be addressed and requested an updated safety assessment, including additional information from clinical studies and adverse-event analyses. The FDA also stated that the product could not be legally marketed until the application received approval. These regulatory requirements highlight the distinction between promising clinical trial findings and the evidentiary and safety standards required for regulatory authorization (Katz, 2004).

The MDMA literature also experienced important research-integrity developments in 2024. Three publications based on phase 2 randomized trials of MDMA-assisted psychotherapy were retracted following concerns regarding protocol violations at a study site. The retraction notices indicated that the violations had not been adequately disclosed and that affected data had not been removed from the analyses. These events do not by themselves determine the validity of the broader MDMA evidence base, particularly because subsequent phase 3 studies were conducted, but they demonstrate the importance of transparent reporting, adherence to approved protocols, appropriate handling of affected data, and independent scrutiny of clinical research findings. They also reinforce the need to distinguish individual study findings from the overall maturity of an evidence base.

Evidence for psilocybin also advanced to phase 3 evaluations in 2025. The COMP005 phase 3 trial of COMP360 psilocybin in treatment-resistant depression reported a statistically significant difference in the primary outcome at 6 weeks, with a mean difference of −3.6 points in change in MADRS scores between the 25-mg and placebo groups (*p* < 0.001) (Compass Pathways plc, 2025). The trial sponsor additionally reported that an independent Data Safety Monitoring Board identified no unexpected safety findings or clinically meaningful imbalance in suicidal ideation between treatment groups. Because these findings were initially reported by the study sponsor, they should be interpreted as emerging phase 3 evidence pending consideration of the full peer-reviewed publication. The results nevertheless represent an important transition from earlier phase 2 evidence toward larger confirmatory trials (Compass Pathways plc, 2025).

The translation of efficacy findings into routine clinical care remains a separate question from efficacy under controlled trial conditions. Recent evidence from the routine treatment of treatment-resistant depression suggests that outcomes observed in clinical practice may differ from those reported in highly selected and closely monitored research populations (Martiadis, 2026). This distinction is particularly relevant for psychedelic-assisted therapy because implementation requires trained therapists, appropriate clinical infrastructure, careful participant selection, preparation and integration procedures, and systems for monitoring adverse psychological and physical effects. Accordingly, evidence from routine care should be considered alongside randomized trials when evaluating the practical generalizability of psilocybin-assisted treatment.

### 4.8 Implications for future research

Several priorities emerged from the mapped evidence. First, adequately powered randomized trials with longer follow-up are needed across compounds and clinical indications, particularly for interventions that currently rely heavily on early-phase or non-peer-reviewed evidence. Second, future studies should more explicitly characterize the psychotherapeutic component, including preparation, therapist orientation, session structure, integration, and treatment fidelity.

Third, methodological studies should continue to address functional unblinding and expectancy through improved comparator conditions and prospective measurement of treatment beliefs. Fourth, safety monitoring should extend beyond acute dosing sessions and incorporate standardized long-term assessment of psychiatric, cardiovascular, and other clinically relevant outcomes.

Finally, studies should examine the interaction between pharmacological and psychotherapeutic components rather than treating PAT as a single indivisible intervention. Designs that can distinguish drug effects, psychological support, expectancy, therapeutic alliance, and contextual effects would provide a stronger basis for understanding how PAT produces clinical change.

### 4.9 Strengths and limitations of the review

This review has several strengths. It mapped both peer-reviewed and relevant non-peer-reviewed evidence, allowing the review to capture not only completed clinical studies but also ongoing trials, regulatory developments, and emerging evidence in a rapidly changing field. The use of the PCC framework and PRISMA-ScR reporting approach also provided a structure appropriate for a heterogeneous evidence base. In addition, the distinction between primary clinical evidence, secondary literature, and contextual sources reduced the risk of treating fundamentally different source types as equivalent.

Several limitations should nevertheless be considered. The search was restricted to English-language sources and covered a limited number of databases. The publication and availability window was restricted to January 2020 through November 2025, meaning that relevant earlier evidence and developments after the search period were outside the scope of the review. The search itself was conducted on 23 February 2026, but no sources published or publicly available after November 2025 were eligible.

The review also did not undertake a formal risk-of-bias assessment or GRADE evaluation. This was consistent with the scoping-review objective of mapping the evidence, but it limits the extent to which the review can make conclusions about comparative efficacy or certainty of treatment effects. Considerable heterogeneity existed across compounds, indications, sample sizes, outcome measures, psychotherapy protocols, and follow-up periods. In addition, overlap between primary studies and secondary reviews creates a possibility of repeatedly encountering the same underlying clinical evidence, although this was addressed during synthesis by distinguishing primary studies from secondary sources.

Finally, publication bias, sponsorship bias, and the inclusion of industry-reported information may have influenced the available evidence, particularly for compounds at earlier stages of clinical development. Non-peer-reviewed sources were therefore treated as contextual or preliminary evidence rather than as equivalent to peer-reviewed clinical findings. These limitations should be considered when interpreting the overall landscape mapped by the review.

## 5 Conclusion

The evidence mapped in this scoping review demonstrates substantial expansion of research into psychedelic-assisted therapy between 2020 and 2025, particularly for MDMA-assisted therapy in PTSD and psilocybin-assisted interventions for depressive disorders. Clinical evidence for LSD-assisted treatment in generalized anxiety disorder also emerged during the review period, whereas DMT-assisted interventions remained at an earlier stage of development and were represented substantially by preliminary or contextual sources.

The evidence does not yet support viewing PAT as a uniform therapeutic intervention. Clinical protocols differ in psychedelic compound, dose, psychological support, therapist approach, preparation, integration, outcome assessment, and follow-up. Methodological challenges, particularly functional unblinding and expectancy effects, further complicate interpretation of treatment effects. Important uncertainties also remain concerning long-term safety, the specific contribution of psychotherapy, and the generalizability of findings from highly selected clinical trial populations.

Future research should therefore move beyond demonstrating whether psychedelic interventions produce clinical change and examine how, for whom, and under what therapeutic conditions such effects occur. Greater standardization and transparent reporting of psychotherapeutic procedures, improved approaches to blinding and expectancy assessment, systematic long-term safety monitoring, and rigorous attention to informed consent, therapist competence, cultural considerations, and equitable access will be important for the responsible development of PAT.

Overall, the field has generated substantial clinical and methodological knowledge but remains characterized by important evidence gaps. Continued rigorous investigation will be necessary to determine the durability, safety, mechanisms, and generalizability of psychedelic-assisted interventions and to establish how emerging findings can be translated appropriately into clinical practice.

## Data Availability

The original contributions presented in the study are included in the article/Supplementary material, further inquiries can be directed to the corresponding author.
