# Supplementary material for: Psychedelic assisted psychotherapy (2020–2025): a scoping review of clinical evidence, mechanisms, safety, and ethics
Source: Front Psychol. 2026 Oct 27;17:1820860. doi: 10.3389/fpsyg.2026.1820860 (PMC13748407; doi:10.3389/fpsyg.2026.1820860)
Supplement: Supplementary file 1 [file Data_Sheet_1.pdf]

Supplementary Table S1. Complete Search Strategies Used for the Scoping Review

Review topic: Psychedelic-assisted therapy and contemporary clinical, psychotherapeutic, methodological, safety, mechanistic, and ethical evidence

Date of database searches: 23 February 2026

Eligibility period: January 2020 to November 2025

Language restriction: English

Information sources: PubMed, PsycINFO, Scopus, and ClinicalTrials.gov

Additional searching: Reference-list screening of relevant publications and reviews

Information source	Date searched		Search strategy	Limits/filters applied
**PubMed**	23 February 2026		(("Psychedelic Agents"[Mesh] OR psychedelic*[tiab] OR "psychedelic-assisted"[tiab] OR "psychedelic assisted"[tiab] OR "psychedelic therapy"[tiab] OR "psychedelic psychotherapy"[tiab] OR "psychedelic-assisted therapy"[tiab] OR "psychedelic-assisted psychotherapy"[tiab]) AND (psilocybin[tiab] OR psilocybe[tiab] OR MDMA[tiab] OR "3,4-methylenedioxymethamphetamine"[tiab] OR methylenedioxymethamphetamine[tiab] OR LSD[tiab] OR lysergide[tiab] OR "lysergic acid diethylamide"[tiab] OR DMT[tiab] OR "N,N-dimethyltryptamine"[tiab] OR dimethyltryptamine[tiab]) AND	English; publication date from 1 January 2020 through 30 November 2025

			(psychotherap*[tiab] OR "psychological support"[tiab] OR "psychological therapy"[tiab] OR "talk therapy"[tiab] OR preparation[tiab] OR integration[tiab] OR "therapeutic support"[tiab] OR "supportive therapy"[tiab]) AND (depress*[tiab] OR "major depressive disorder"[tiab] OR MDD[tiab] OR PTSD[tiab] OR "post-traumatic stress disorder"[tiab] OR anxi*[tiab] OR "substance use disorder"[tiab] OR addiction[tiab] OR psychiatric*[tiab] OR mental[tiab]))	
**PsycINFO**	23 February 2026		((DE "Psychedelic Drugs" OR TI,AB(psych* N3 (psychedelic* OR "psychedelic-assisted" OR "psychedelic assisted" OR "psychedelic therapy" OR "psychedelic psychotherapy")) OR TI,AB("psychedelic-assisted therapy") OR TI,AB("psychedelic-assisted psychotherapy")) AND (TI,AB(psilocybin OR psilocybe OR MDMA OR "3,4-methylenedioxymethamphetamine" OR methylenedioxymethamphetamine OR LSD OR lysergide OR "lysergic acid diethylamide" OR DMT OR "N,N-dimethyltryptamine" OR dimethyltryptamine)) AND (TI,AB(psychotherap* OR "psychological support" OR	English; publication date from 1 January 2020 through 30 November 2025

			"psychological therapy" OR preparation OR integration OR "therapeutic support" OR "supportive therapy")) AND (TI,AB(depress* OR "major depressive disorder" OR PTSD OR "post-traumatic stress disorder" OR anxi* OR "substance use disorder" OR addiction OR psychiatric* OR mental)))	
**Scopus**	23 February 2026		TITLE-ABS-KEY((psychedelic* OR "psychedelic-assisted" OR "psychedelic assisted" OR "psychedelic therapy" OR "psychedelic psychotherapy" OR "psychedelic-assisted therapy" OR "psychedelic-assisted psychotherapy") AND (psilocybin OR psilocybe OR MDMA OR "3,4-methylenedioxymethamphetamine" OR methylenedioxymethamphetamine OR LSD OR lysergide OR "lysergic acid diethylamide" OR DMT OR "N,N-dimethyltryptamine" OR dimethyltryptamine) AND (psychotherap* OR "psychological support" OR "psychological therapy" OR preparation OR integration OR "therapeutic support" OR "supportive therapy") AND (depress* OR "major depressive disorder" OR MDD OR PTSD OR "post-traumatic stress disorder" OR anxi* OR "substance use	English; publication year 2020–2025

			disorder" OR addiction OR psychiatric* OR mental))	
**ClinicalTrials.gov**	23 February 2026		(psilocybin OR MDMA OR methylenedioxymethamphetamine OR LSD OR lysergide OR DMT OR N,N-dimethyltryptamine) AND (psychedelic-assisted OR psychedelic OR psychotherapy OR psychological support OR therapy)	Studies first posted or publicly available from January 2020 through November 2025; records relevant to human clinical research
**Reference-list screening**	23 February 2026 onward		Reference lists of included primary studies and relevant systematic, narrative, methodological, and mechanistic reviews were manually screened for additional potentially relevant sources.	No additional database-specific filters; identified sources were assessed against the predefined eligibility criteria.

Supplementary Table S2. Methodological, Ethical, Safety, and Implementation Findings in Psychedelic-Assisted Therapy

Domain	Key finding	Implication
**Blinding and expectancy**	Functional unblinding is a recurring challenge because psychedelic effects can reveal treatment allocation	Future trials should assess treatment-guessing and expectancy prospectively
**Psychotherapeutic variability**	Preparation, support and integration vary substantially across studies	Psychotherapy should be described and reported more consistently
**Therapist training**	Therapist competence and experiential/professional training are important considerations	Training standards require clearer empirical definition
**Acute safety**	Headache, nausea, dizziness, anxiety and transient cardiovascular effects were reported	Appropriate screening, supervision and monitoring remain necessary
**Long-term safety**	Follow-up periods and sample sizes vary considerably	Longer-term systematic monitoring is required
**Informed consent**	Altered states may affect vulnerability and the consent environment	Consent procedures should clearly address the psychedelic experience and potential risks
**Professional boundaries**	Therapeutic touch and interpersonal boundaries require explicit safeguards	Clear professional standards and procedures are needed
**Equity**	Cost, availability, geographic access and representation may affect access	Future research and implementation should address accessibility and representation
**Cultural considerations**	Psychedelic practices may intersect with Indigenous and cultural traditions	Cultural context, representation and benefit-sharing should be considered
