# Supplementary material for: Psychedelic assisted psychotherapy (2020–2025): a scoping review of clinical evidence, mechanisms, safety, and ethics
Source: Front Psychol. 2026 Oct 27;17:1820860. doi: 10.3389/fpsyg.2026.1820860 (PMC13748407; doi:10.3389/fpsyg.2026.1820860)
Supplement: Supplementary file 2 [file Data_Sheet_2.docx]

| **Section and Topic** | **Item #** | **Checklist item** | **Location where item is reported** |
| --- | --- | --- | --- |
| **TITLE** | | |  |
| Title | 1 | Psychedelic Assisted Psychotherapy (2020–2025): A Scoping Review of Clinical Evidence, Mechanisms, Safety, and Ethics | 01 |
| **ABSTRACT** | | |  |
| Abstract | 2 | **Background/Rationale:** Rapid growth in psychedelic-assisted psychotherapy research shows promising clinical outcomes for PTSD and major depressive disorder, but relapse, limited response, contextual influences (“set and setting”), and ethical considerations require systematic synthesis.  **Objectives:** To characterize current PAT models, summarize clinical outcomes, and identify shared principles and divergences across protocols.  **Methods:** Structured search of PubMed, PsycINFO, Scopus, and ClinicalTrials.gov for clinical studies (January 2020–November 2025) pairing psilocybin, MDMA, LSD, or DMT with structured psychotherapy.  **Results:** 3,264 records identified; 19 studies included. Strongest efficacy signals observed for MDMA-assisted therapy in PTSD and psilocybin-assisted therapy in depression. Safety generally acceptable; inconsistencies remain in adverse-event reporting and long-term follow-up.  **Conclusions:** Future priorities include psychotherapy manual standardization, improved blinding and expectancy controls, strengthened adverse-event monitoring, and robust ethical safeguards. | 01-02 |
| **INTRODUCTION** | | |  |
| Rationale | 3 | The review is justified by the rapid reemergence of psychedelic-assisted therapy after decades of limited regulatory activity, accompanied by encouraging phase 2 and phase 3 findings demonstrating clinically meaningful improvements in post-traumatic stress disorder and major depressive disorder. At the same time, significant methodological challenges persist, including difficulties with effective blinding and the influence of expectancy effects, which complicate interpretation of outcomes. Regulatory authorities have raised concerns about distinguishing the pharmacological effects of psychedelic compounds from the contributions of psychotherapy and contextual factors. Research is also expanding into additional indications such as anxiety disorders, substance use disorders, and burnout-related depressive symptoms. Alongside this growth, important ethical considerations have emerged, including safeguarding informed consent during altered states of consciousness, maintaining appropriate therapist–participant boundaries, promoting equitable access, and responsibly engaging with Indigenous knowledge systems. Taken together, this rapidly evolving clinical and regulatory landscape underscores the need for a comprehensive synthesis of current evidence, models, and ethical frameworks. | 02 |
| Objectives | 4 | The review aims to:   1. Characterize current psychedelic-assisted therapy models 2. Summarize reported clinical outcomes 3. Examine safety findings 4. Identify ethical and mechanistic themes 5. Delineate shared principles and divergences across protocols 6. Identify priorities for future research and implementation | 01 |
| **METHODS** | | |  |
| Eligibility criteria | 5 | **Inclusion Criteria:**   - Peer-reviewed human clinical trials or systematic reviews - Published January 2020–November 2025 - Use of psilocybin, MDMA, LSD, or DMT - Combined with structured psychotherapy - Psychiatric indications (e.g., PTSD, depression, anxiety, substance use) - English language   **Exclusion Criteria:**   - Preclinical/animal studies - Pharmacology-only studies without psychotherapy - Editorials/opinion pieces - Non–peer-reviewed literature - Recreational/non-clinical psychedelic use - Non-English publications   **Grouping for Synthesis:**  Studies grouped by:   - Psychedelic compound - Clinical indication - Ethical/mechanistic focus | 03 |
| Information sources | 6 | Databases searched:   - PubMed - PsycINFO - Scopus - ClinicalTrials.gov   Additional:   - Reference lists of included studies and relevant reviews   Search timeframe:  January 2020 – November 2025 | 03 |
| Search strategy | 7 | - Combination of controlled vocabulary and free-text terms - Core terms included: - psychedelic-assisted therapy - psilocybin - MDMA - LSD - DMT - clinical trial - psychiatry - psychotherapy - ethics. | 03-04 |
| Selection process | 8 | - Two reviewers independently screened titles and abstracts - Full-text articles assessed independently - Discrepancies resolved by discussion   Flow of records:   - 3,693 records identified - 812 duplicates removed - 2,760 screened - 121 full texts assessed - 15 studies included | 04 |
| Data collection process | 9 | - Two reviewers extracted data independently - Extracted variables included: - Study design - Participant characteristics - Psychedelic agent - Therapeutic protocol - Clinical indication - Primary outcomes - Safety/adverse events - Ethical/mechanistic findings | 04-05 |
| Data items | 10a | Outcomes included:   - Clinical symptom reduction (e.g., PTSD, depression scores) - Safety outcomes (adverse events) - Long-term follow-up findings - Mechanistic correlates of response - Ethical considerations related to delivery   All reported clinical outcomes relevant to psychiatric endpoints were considered. | 05 |
|  | 10b | - Participant demographics - Study design characteristics - Type of psychotherapy model - Dosing structure - Regulatory context - Ethical safeguards - Mechanistic constructs (e.g., subjective experience quality)   No explicit assumptions about missing data described. | 05 |
| Study risk of bias assessment | 11 | No formal risk-of-bias assessment tool was applied. This is consistent with scoping review methodology, the review mapped evidence descriptively without quality grading. | Not reported (no risk-of-bias assessment described). |
| Effect measures | 12 | No pooled quantitative synthesis conducted. Effect measures (e.g., symptom scale reductions) were reported as presented in original studies but not statistically combined. | 06 |
| Synthesis methods | 13a | Studies were categorized by:   - Psychedelic compound - Clinical indication - Thematic focus (efficacy, safety, ethics, mechanisms) | 05-08 |
|  | 13b | No statistical conversions or imputation procedures performed. Data summarized as reported. | Not reported (no statistical synthesis methods described). |
|  | 13c | - Narrative synthesis - Flow diagram (Figure 1) - Tabular summaries of included studies. | 05-06 |
|  | 13d | Narrative synthesis selected due to:   - Heterogeneity in interventions - Variation in psychotherapy models - Differences in outcome measures - Inclusion of ethics/mechanistic reviews   No meta-analysis performed. | Not reported (rationale for synthesis approach not explicitly stated). |
|  | 13e | No formal subgroup analysis or meta-regression performed. | Not reported. |
|  | 13f | No sensitivity analyses conducted. | Not reported. |
| Reporting bias assessment | 14 | No formal assessment (e.g., funnel plots) conducted due to absence of meta-analysis | Not reported. |
| Certainty assessment | 15 | No formal certainty grading (e.g., GRADE) performed, consistent with scoping review objectives to map literature rather than determine comparative effectiveness. | Not reported. |
| **RESULTS** | | |  |
| Study selection | 16a | The literature search (2020–2025) identified randomized controlled trials, phase 2 and 3 studies, and relevant regulatory or methodological publications evaluating MDMA, psilocybin, LSD (MM120), and DMT (SPL026) for PTSD, major depressive disorder (MDD), treatment-resistant depression (TRD), and anxiety disorders.  After removal of duplicates and screening of titles and abstracts, full texts were assessed for eligibility. Studies were included if they:   - Investigated psychedelic-assisted therapy (PAT) in a clinical psychiatric population - Included structured psychotherapy alongside drug administration - Reported clinical efficacy and safety outcomes - Were published between 2020–2025   Early-phase safety-only studies without clinical outcomes, non-controlled observational reports, and non-psychiatric indications were excluded.  The final synthesis included:   - Two phase 3 trials of MDMA-assisted therapy for PTSD - One phase 2 trial of psilocybin for MDD - One phase 2b trial of psilocybin for TRD - One phase 2b trial of LSD (MM120) for generalized anxiety disorder - One phase 2a trial of DMT (SPL026) for MDD - Supporting follow-up, regulatory, and mechanistic publications   A PRISMA flow diagram would illustrate identification, screening, eligibility, and inclusion stages. | 04 |
|  | 16b | Studies excluded after full-text review included:   - Phase 1 safety studies in healthy volunteers (e.g., early DMT tolerability trials) due to absence of clinical efficacy outcomes - Observational extensions without comparator arms when primary randomized data were already included - Studies lacking structured psychotherapy components - Commentaries or narrative reviews without original outcome data   Exclusions were based on failure to meet predefined clinical efficacy and methodological criteria. | 04 |
| Study characteristics | 17 | Included studies were multicenter randomized controlled trials conducted primarily in the United States and Europe. Key characteristics:  **PTSD — MDMA-assisted therapy**   - Population: Severe PTSD - Design: Phase 3, randomized, placebo-controlled - Intervention: MDMA + manualized psychotherapy - Comparator: Placebo + identical psychotherapy - Primary outcome: CAPS-5 - Follow-up: Up to several months post-treatment   **MDD — Psilocybin**   - Population: Moderate-to-severe MDD - Design: Phase 2, randomized - Intervention: 25 mg psilocybin + structured support - Primary outcome: Depression severity (e.g., MADRS) - Follow-up: 4 weeks   TRD — Psilocybin   - Population: Treatment-resistant depression - Design: Phase 2b, randomized dose-response - Intervention: 1 mg vs 10 mg vs 25 mg psilocybin - Primary outcome: MADRS change at 3 weeks - Follow-up: Up to 12 weeks; extension to 52 weeks in subgroup   GAD — LSD (MM120)   - Population: Generalized anxiety disorder - Design: Phase 2b randomized - Intervention: 25–200 µg doses (100 µg most effective) - Primary outcome: HAM-A at 4 weeks - Follow-up: 12 weeks - **MDD — DMT (SPL026)** - Population: Moderate-to-severe MDD - Design: Phase 2a, two-stage - Intervention: Single IV DMT dose + supportive therapy - Primary outcome: Depression severity at 2 weeks - Follow-up: 12 weeks - Psychotherapy ranged from manualized supportive therapy to integrative trauma-focused models. | 04 |
| Risk of bias in studies | 18 | Common risk of bias domains:   - **Randomization**: Generally adequate in phase 2–3 trials - **Allocation concealment**: Reported in large multicenter trials - **Blinding**: High risk of functional unblinding due to psychoactive effects - **Outcome assessment**: Clinician-rated scales; some trials used blinded raters - **Attrition**: Generally low to moderate - **Selective reporting**: Limited evidence but reliance on sponsor-led trials noted   Primary risk across studies: expectancy effects and compromised blinding. | Not reported (no formal risk-of-bias results presented). |
| Results of individual studies | 19 | **MDMA for PTSD**   - Effect size: d ≈ 0.91 (large) - CAPS-5 change: −23.7 vs −14.8 (MDMA vs placebo) - Sustained benefit at follow-up - Mild-to-moderate transient adverse events   **Psilocybin for MDD**   - Rapid symptom reduction within 24 hours - Sustained improvement to 4 weeks - Clinically meaningful effect size   **Psilocybin for TRD**   - Dose-response relationship - 25 mg superior to 1 mg - Higher remission rates at 3 weeks - Sustained response up to 12 weeks   **LSD (MM120) for GAD**   - Significant HAM-A reduction at 4 weeks - Dose-response effect - High response/remission rates   **DMT (SPL026) for MDD**   - Rapid antidepressant effect within 1 week - Primary endpoint met at 2 weeks - Durable effects in subset at 12 weeks   Across studies, serious adverse events were rare (~4%). | 08 |
| Results of syntheses | 20a | Syntheses primarily included phase 2–3 randomized trials with moderate sample sizes. Functional unblinding was a consistent methodological limitation. | 05-08 |
|  | 20b | Formal meta-analysis was not conducted due to:   - Heterogeneity of compounds - Different psychiatric indications - Variable dosing regimens - Differences in psychotherapy models   Direction of effect across trials consistently favored psychedelic-assisted therapy over control. | Not reported. |
|  | 20c | Sources of heterogeneity included:   - Psychotherapy modality - Dose variation - Baseline severity - Prior treatment resistance - Expectancy and blinding integrity | Not reported. |
|  | 20d | Individual trials conducted per-protocol and intention-to-treat analyses; results were generally robust across analytic approaches. | Not reported. |
| Reporting biases | 21 | Most pivotal trials were industry-sponsored. While primary outcomes were consistently reported, long-term negative findings and unpublished data remain possible sources of reporting bias. Trial registration mitigates but does not eliminate this risk. | Not reported. |
| Certainty of evidence | 22 | Using GRADE principles:   - MDMA for PTSD: High certainty - Psilocybin for TRD: Moderate-to-high certainty - Psilocybin for MDD: Moderate certainty - LSD for GAD: Moderate (phase 2b) - DMT for MDD: Low-to-moderate (early phase)   Downgrading factors included functional unblinding, limited long-term data, and psychotherapy variability | Not reported. |
| **DISCUSSION** | | |  |
| Discussion | 23a | The strongest evidence supports MDMA-assisted therapy for severe PTSD and psilocybin-assisted therapy for depressive disorders. Early-phase LSD and DMT trials show promise but require replication. Compared with conventional pharmacotherapy, PAT demonstrates rapid onset and sustained benefit after limited dosing sessions. | 08 |
|  | 23b | - Small sample sizes in early-phase trials - Limited diversity in participant populations - Short follow-up duration - Heterogeneous psychotherapy models - Functional unblinding | 08-11 |
|  | 23c | - No quantitative meta-analysis - Reliance on published reports - Potential publication bias - Variability in reporting of adverse events | Not reported. |
|  | 23d | Psychedelic-assisted therapy (PAT) could be a promising option for people with hard-to-treat psychiatric conditions, especially when delivered in controlled settings with proper preparation, supervision, and follow-up support. For policy, it is important to ensure therapists have adequate training, adverse events receive close monitoring, and ethical safeguards protect patients during these intense experiences. Research should focus on larger phase 3 trials to confirm results, clear and consistent psychotherapy manuals, long-term safety records, and better methods to reduce expectancy effects and maintain blinding in studies. | 09-10 |
| **OTHER INFORMATION** | | |  |
| Registration and protocol | 24a | This review was not prospectively registered. | Not reported. |
|  | 24b | A formal protocol was not published prior to conducting the review. | Not reported. |
|  | 24c | Not applicable. | Not applicable / Not reported. |
| Support | 25 | Deanship of Graduate Studies and Scientific Research at Qassim University for financial support (QU-APC-2026). | 12 |
| Competing interests | 26 | The authors declare no competing interests | 12 |
| Availability of data, code and other materials | 27 | No new datasets were generated. All data were derived from publicly available published clinical trials. Extracted summaries and analytic notes are available from the corresponding author upon reasonable request. | 11 |

*From:*  Page MJ, McKenzie JE, Bossuyt PM, Boutron I, Hoffmann TC, Mulrow CD, et al. The PRISMA 2020 statement: an updated guideline for reporting systematic reviews. BMJ 2021;372:n71. doi: 10.1136/bmj.n71. This work is licensed under CC BY 4.0. To view a copy of this license, visit <https://creativecommons.org/licenses/by/4.0/>
